# A Mark on the Skin, a Lesson for the Memory

**DOI:** 10.4269/ajtmh.25-0600

**Published:** 2026-02-03

**Authors:** Ángel Sebastián Rodríguez-Pazmiño

**Affiliations:** One Health Research Group, Universidad de Las Américas, Quito, Ecuador

I still have a scar on my leg, a reminder of an infection I got after visiting egg-laying chicken farms in Tungurahua, Ecuador in 2019. I never imagined that the experience would not only leave a mark on my skin but also teach me a lesson about vulnerability and commitment.

One day, I received a message from a friend from my days as an animal rights activist. She invited me to a workshop in Quito held by the Gallinas Libres (Free Hens) project, part of the international coalition Open Wing Alliance. The session focused on chicken behavior and welfare. “The first day will be theoretical, and the next day we’ll go on an interesting trip,” she told me. There, I learned that chickens form complex social structures, can remember more than a hundred individuals (including humans), and are (no less) direct descendants of dinosaurs.

The next day, we set off for Tungurahua Province to visit egg-laying hen farms, document the hens’ living conditions, and film material for a short documentary aimed at raising public awareness. Our group included two Ecuadorians, one Brazilian, one Chilean, and one Argentinean. The long drive left no room for boredom. Between farm visits, we swapped stories about our countries and the work that we each did for animals. We also reflected on what it takes to sustain activism in Latin America, where social priorities, limited institutional support, and cultural and regulatory barriers often make animal welfare easy to overlook.

The first farm was near the road to the city of Baños, surrounded by mountains covered with subtropical vegetation. A rectangular building approximately 80 meters long and 10 meters wide housed hundreds of birds arranged in rows of multistory metal cages. The smell of feces was suffocating, and the noise, at times, was deafening. The hens looked exhausted, with little room to move; some ended up on top of their companions. We observed their clipped beaks, which the worker explained served to prevent them from killing each other. Occasionally, a rooster that had infiltrated the flock since they were chicks would appear, a curious detail amid so much uniformity.

The second farm was even larger, almost like a small coliseum filled with cages on several floors. There, we talked more with the workers, humble people from rural areas who told us that they enjoyed their work, even though it was very demanding. One said he did it because he liked “caring for and loving the animals.” Upon hearing this, our Brazilian friend made a gesture that said “yeah, right.”

To enter the main area where the chicken coops were located, we had to ask the site manager for permission. She was a little reluctant at first, but after explaining that we had come from far away and cracked a few jokes to break the ice, she let us in. At that point, we were able to discuss various topics related to her job with her. She expressed concern that the business can sometimes be unprofitable when chicken feed becomes expensive, especially when there are no government subsidies for corn. While we talked, my colleagues from Chile and Argentina recorded and took pictures of the chickens and the facilities.

During the workshop, we had learned that a well-cared-for hen can live 8–12 years, lay eggs regularly for 2 or 3 of those years, and remain a social and active animal afterward. On intensive farms, however, their lives rarely last beyond 2 years. So, while we were on this farm, I wanted to know what happened when a chicken got sick. Here, a worker explained that when hens fall ill or begin to age and production declines, they are slaughtered. “Nothing is wasted in this business,” he said; their bodies become soup flavorings, dog food, or fertilizer.

The site manager on this second farm explained that treating sick birds was not profitable: “The cost of the medication exceeds the value of what the chicken produces in eggs. Weak, injured, or unproductive birds are removed from the cages and slaughtered.” As we were leaving, we saw large bins with the bodies of the discarded birds. It was a shocking scene.

A few days later back in Ambato, my hometown, I began to notice small pustules on my leg. At first, I thought they were just pimples, something temporary. Over time, they grew, multiplied, and spread to other parts of my body, including my chest. Some reached several centimeters in diameter, and I developed a high fever. When I visited a dermatologist, he prescribed antibiotics and some topical creams. After weeks of treatment, I improved, although the scars remained. I never knew for sure which micro-organism had infected me, but later, after reviewing information on the internet, I suspected it was a *Staphylococcus aureus* infection.

That experience occurred during a period of personal introspection when activism gave me purpose. The scars remind me that science can (and should) be linked to ethics and compassion. However, this often presents me with a dilemma. In Ecuador, eggs are a staple food, forming part of the daily diet of millions of people. That reality coexists with another; intensive production models encourage the spread of respiratory diseases among birds, some of which have demonstrated pandemic potential throughout history. The paradox is evident; although this food nourishes families, the system that produces it poses risks to both animal and human health.

The lessons from that trip to Tungurahua Province near the city of Baños are connected to the laboratory where I now work. We analyze the circulation of bacteria and viruses in chickens, guinea pigs, dogs, cats, cows, wild animals, and humans. Here, the concept of One Health (the interconnection between human, animal, and environmental health) is central. Whenever I have the opportunity, I try to add another: animal welfare; it reminds me, like the scar on my leg and the Gallinas Libres project, that all action must be geared toward protecting the most vulnerable beings (human or nonhuman) if we aspire to a more just and sustainable world.

